# ParMap, an algorithm for the identification of small genomic insertions and deletions in nextgen sequencing data

**DOI:** 10.1186/1756-0500-3-147

**Published:** 2010-05-27

**Authors:** Hossein Khiabanian, Pieter Van Vlierberghe, Teresa Palomero, Adolfo A Ferrando, Raul Rabadan

**Affiliations:** 1 Department of Biomedical Informatics, Columbia University College of Physicians and Surgeons, New York, NY, USA; 2 Center for Computational Biology and Bioinformatics, Columbia University College of Physicians and Surgeons, New York, NY, USA; 3 Institute for Cancer Genetics, Columbia University College of Physicians and Surgeons, New York, NY, USA; 4 Department of Pathology, Columbia University College of Physicians and Surgeons, New York, NY, USA; 5 Department of Pediatrics, Columbia University College of Physicians and Surgeons, New York, NY, USA

## Abstract

**Background:**

Next-generation sequencing produces high-throughput data, albeit with greater error and shorter reads than traditional Sanger sequencing methods. This complicates the detection of genomic variations, especially, small insertions and deletions.

**Findings:**

Here we describe ParMap, a statistical algorithm for the identification of complex genetic variants, such as small insertion and deletions, using partially mapped reads in nextgen sequencing data.

**Conclusions:**

We report ParMap's successful application to the mutation analysis of chromosome X exome-captured leukemia DNA samples.

## Background

One of the major technological advances in biology in the last few years has been the development of high throughput nextgen sequencing systems that produce gigabases of data in a single run, and allow an unbiased view of the whole genome without relying on prior knowledge about the disease-causing alterations. These ultradeep sequencing technologies produce large amounts of sequence data, which increase the sequencing depth and allow for better statistics in calling various genomic variations. However, they do so at the cost of reducing the read length and increasing the error rate relative to traditional Sanger sequencing. Thus, the development of efficient statistical and computational methods for the high confidence call of genomic variants is needed for the analysis of these high throughput datasets.

At this point, the detection of single mutations and large copy number variations using deep sequencing data is fairly straight forward [1,2], whereas the identification of small (less than 10 nucleotides) insertions and deletions is more challenging. A few algorithms have been developed for detecting complex genomic variants, such as structural variations and insertions and deletions, using mate-pair or paired-end reads [3,4], however, identifying small insertion/deletions in fragment (single-end) data has proved to be very difficult. Mapping algorithms that are designed for very short reads have to assign large penalties for introducing gaps in the middle of the alignment in order to map the majority of the reads efficiently. Conversely, decreasing the gap penalties, increases the number of reads that are mapped with low confidence. However, these methods can partially map reads to a reference genome with gaps at the either end, without significantly reducing the alignment score. Although these gaps may be caused by systematic errors in the sequencing and mapping processes, we hypothesized that gaps that appear in multiple reads at a given position on the genome may reflect the presence of a more complex genomic variant such as insertion, deletion, or multiple base changes. However, this algorithm is not capable of detecting structural variants.

**Figure 1:**
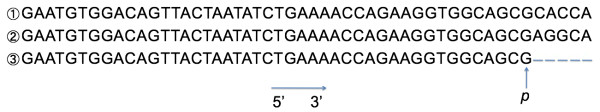
**ParMap employs reads that are partially mapped to a reference genome to identify genomic variations**. These variations include small insertions and deletions of less than 10 nucleotides. When the sequenced read (line 2) is mapped to the reference genome (line 1), the unmatched bases are marked as gaps (line 3), adjacent to position *p *(Methods).

**Figure 2:**
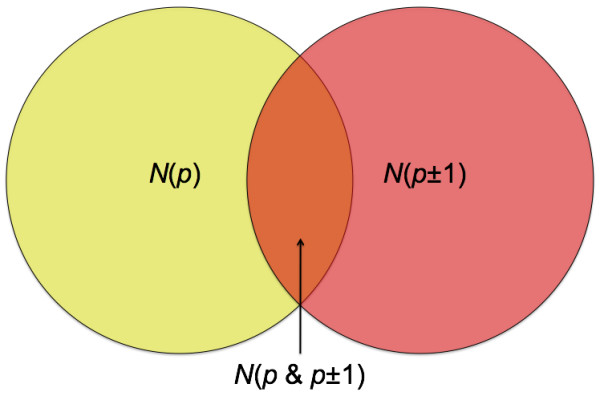
**A measure based on the number of reads that only cover position *p *without covering its neighboring position (*p *± 1) in the direction of the gap is calculated**. In other words, we find the ratio of the intersection (orange area) and the union (yellow and red areas) of the two sets of reads that cover *p *or *p *± 1 (Figure 1). Here, *N*(*p*), *N*(*p *± 1), and *N*(*p *&*p *± 1) are the number of reads that cover position *p, *position *p *± 1, and both positions, respectively (Methods).

Following this principle, we aimed to develop a procedure for identifying small genomic insertions and deletions with high confidence and built an algorithm (ParMap) capable of producing a list of candidates (along with their nucleotide sequence), through statistical analysis of partially mapped reads (Figure 1). Specifically, ParMap calculates a measure based on the number of reads that only cover the positions adjacent to a gap without covering their neighboring positions in the direction of the gap, to identify the possible locations of genomic insertions or deletions (Figure 2 and Methods).

## Results and discussion

To test the ability of this method to detect novel complex genomic variants, such as insertion, deletion, and multiple base changes, we applied ParMap to the analysis of SOLiD 3 chromosome X exome sequencing data from 12 T-cell acute lymphoblastic leukemia (T-ALL) DNA samples [5]. In this experiment leukemia DNA samples were fragmented and ligated to adapters to generate SOLiD sequencing libraries, which were amplified and subsequently enriched in chromosome X exonic sequences using the SureSelect Target Enrichment System [6], a platform which targets 5,217 exonic regions encompassing 3,045,708 nucleotides in the X chromosome. The Chromosome X exome captured DNA samples were sequenced with the Applied Biosystems SOLiD 3 platform using 1/8^th ^of sequencing slide per sample to produced a total of 105,302,787 fifty-base long fragment reads. The SOLiD platform employs a ligation based chemistry and a two-base encoding system, where each pair of nucleotides is reported with a different color, depending on the first base within the pair. Therefore, to call a single-base change relative to the reference sequence in nucleotide-space, two consecutive color-space mismatches must be observed. Single color-space mismatches solely report errors in the reads [7].

To ensure an optimum mapping of these sequencing results, we created a reference sequence containing all chromosome X capture targeted regions plus adjacent 50 flanking bases using the March 2006 human reference sequence assembly (hg18). We used the SHRiMP algorithm with its default parameters for mapping the reads [8]. For further analysis of our dataset, we only included the reads with a maximum number of two color-space mismatches that were uniquely mapped to the reference genome (approximately 31% of the raw reads). An average 90.1% of the reference genome was found covered in the samples, with a mean depth of 42 per base. Less restrictive filtering increased the false positive rate of candidate genomic variants without a significant increase in the coverage.

We created a candidate list of single-base variants for which a minimum of 3 reads (consistent with 1% estimated error rate of this particular run) should map to the candidate's position, with more than 75% of them calling the nucleotide change. T-ALL samples such as the ones analyzed in this series contain over 80% tumor cells, however a small fraction of contaminating normal cells is expected. Because of the possibility of this contamination, we did not enforce a 100% consensus among the reads. To identify genetic alterations with the most direct impact in gene function we focused on the analysis of non-synonymous changes in the captured exons.

In this analysis we noticed numerous systematic errors that cannot be corrected by increasing the sequencing depth, i.e. genomic variants that are reported systematically beyond the statistical expectations from the estimated error rates. These systematic errors arise from pre-sequencing operations, ligation-based sequencing, mapping, and specific genomic variants in the reference genome. Due to these redundancies in the capture or mapping, some variations appear in most of the samples. For instance, an identical single-base variant in the exonic region of the SLC6A8 gene was found in 10 samples, within 73% of the reads, beyond the statistical expectations from the estimated error rates. Therefore, we removed the positions that seemed prone to systematic errors by combining the candidate lists from all the samples and only keeping the genomic variations that occur in less than 3 samples at a given position. None of the variations that were found in more than 3 samples were validated via Sanger sequencing.

Within the 12 samples analyzed, we identified 66 exonic non-synonymous single-base variant candidates, which were not listed as already known polymorphisms in the human genome [9]. Overall, 61/66 (92%) of these candidates were confirmed via Sanger sequencing (Table 1). Applying the same filtering criteria results in a similar number of candidates when another alignment algorithm, Corona-Lite [10], is run on our dataset. This indicates that the number of significant candidates does not depend on the mapping method.

**Table 1:** Summary of the efficiency of our identification methods.

	Number of Genomic Variation Candidates	Number of Confirmed Candidates	Percentage
Singe-base Analysis	66	61	**92%**

ParMap	7	4	**57%**

Total	73	65	**89%**

In total, 89% of our candidates were confirmed via Sanger sequencing.

Next, we applied the ParMap algorithm to our dataset to identify possible more complex variants such as small insertions and deletions. Following on the selection of candidate variants using ParMap filtering criteria, we selected the ones that were detected in a single sample each. In this analysis, we found a high prevalence of systematic errors in intron-exon boundaries, which may reflect impaired ligation-based sequencing in these positions. Therefore, candidate variants located in intron-exon boundaries were discarded and excluded from further analysis. ParMap identified a total of 7 candidate complex variants (Table 1). Using Sanger sequencing of PCR products encompassing these sequences, we confirmed four indels in four different samples, including two genomic deletions of 3 and 6 nucleotides and two genomic insertions of 5 and 3 nucleotides. Notably, the genomic sequences identified in each of these two insertions matched the predicted sequence variant in the ParMap's results.

## Conclusions

We have demonstrated the successful identification of high confidence genomic variants in nextgen sequencing data using a combination of single nucleotide analysis and ParMap. (Currently, ParMap is available for download upon request from the authors.) Overall, 89% of our candidate variants were experimentally validated in this series. ParMap may enhance the identification of elusive complex genetic variants such as small insertions and deletions in nextgen sequencing data, taking advantage of partially mapped reads that might otherwise be discarded.

## Methods

In addition to the completely mapped reads and reads reporting single-base changes, the dataset includes partially mapped reads, with the unmatched positions marked as gaps. These reads either start or end with a gap region that is as long as 20% of the length of the read (Figure 1). ParMap makes use of such reads and for any position *p *that is adjacent to a gap region and is not the starting or ending position of an exon, calculates the following quantities:

1. *N*(*p*): The number of reads that cover position *p*.

2. *N*(*p *± 1): The number of reads that cover position *p *± 1. (Plus, if *p *is the position after the gap and minus, if *p *is the position before the gap, in the direction of the positive strand.)

3. *N*(*p *&*p *± 1): The number of reads that cover both positions of *p *and *p *± 1.

We define

which is an inverse measure of the number of reads that only cover the position *p *without covering its neighboring position in the direction of the gap (Figure 2). Therefore, the smaller the value of *r*, the higher the chance for the gap to be due to a real change in the sequenced genome. Moreover, because the reads in which position *p *is adjacent to a gap region are already collected, referring back to each read prior to the mapping, the genomic sequence of the gap region can be extracted.

We apply the following criteria to produce a list of candidates: the value of *r *should be less that 0.35 and at least 5 reads should map to *p *adjacent to a gap, reporting a consensus sequence for it. To reduce the systematic errors due to mapping artifacts, we remove the candidates whose gap regions cover the already known polymorphisms of the human genome. We experimentally observed that less restrictive criteria increased the number of false positives.

## Competing interests

The authors declare that they have no competing interests.

## Authors' contributions

HK and RR conceived the ParMap algorithm. HK coded the algorithm and applied it to high throughput sequencing data obtained by TP at the Genomics Technologies Shared Resource of Columbia University. Validation was performed by PVV in AF laboratory. All authors contributed to the development of the method and wrote the paper.
